# Transitioning Towards Sustainable Diets: Pulses, Their Role in Modulating Gut Microbiota and Metabolic Health

**DOI:** 10.3390/nu18152571

**Published:** 2026-08-06

**Authors:** Claudia E. Lazarte, Rucha Rane, Frida Hållenius, Olena Prykhodko

**Affiliations:** 1 National Food Institute, Technical University of Denmark (DTU), 2800 Kongens Lyngby, Denmark; 2 Department of Process and Life Science Engineering, Lund University, 221 00 Lund, Sweden; rucha.rane@ple.lth.se (R.R.); frida.hallenius@ple.lth.se (F.H.); olena.prykhodko@ple.lth.se (O.P.)

**Keywords:** pulses, gut microbiota, metabolic health, sustainability

## Abstract

**Background:** The global shift to sustainable diets has raised interest in plant-based protein sources, particularly pulses, due to their nutritional value, health benefits, and low environmental impact. Pulses are rich in protein, dietary fiber, and a variety of bioactive compounds that may influence gut microbiota and metabolic health. **Objective:** This narrative review aims to evaluate the role of pulse consumption in modulating gut microbiota, improving metabolic health, and contributing to the prevention of non-communicable diseases (NCDs), while considering the impact of processing techniques such as fermentation and sustainability aspects. **Methods:** A comprehensive literature search was conducted using major scientific databases to identify relevant studies published during the last 10 years. Evidence from in vitro, in vivo experimental and human studies was included, focusing on pulse composition, bioactive compounds, gut microbiota modulation, metabolic outcomes, and the effects of processing methods such as fermentation. **Results:** Pulses demonstrate promising potential to improve gut health by promoting beneficial microbiota, increasing short-chain fatty acid production, and enhancing intestinal barrier function. Evidence also suggests favorable effects on lipid metabolism, glycemic control, and inflammation. Processing techniques, particularly fermentation, improve nutrient bioavailability, digestibility, and bioactive profiles. Beyond health outcomes, this review places pulses in the larger context, emphasizing their contribution to healthy, sustainable diets. **Conclusions:** Pulses are key components for achieving sustainable, health-promoting diets with promising effects on gut and metabolic health. However, current evidence is largely derived from preclinical studies, highlighting the need for long-term human interventions and scalable processing strategies to fully realize their potential.

## 1. Introduction

Pulses, defined as the dried edible parts of legumes, have been consumed globally for centuries and remain essential components of many traditional diets. For this review, the term ‘pulses’ is used to encompass both pulses and legumes. In recent decades, pulses have gained renewed attention due to their role in the transition toward sustainable dietary patterns. This is largely attributed to their favorable nutritional profile, characterized by high content of plant protein, dietary fiber, and diverse phytonutrients, alongside environmental advantages such as lower greenhouse gas emissions, reduced land use, and contributions to soil fertility compared with many animal-based protein sources [1]. These attributes position pulses at the forefront of sustainable and health-promoting food systems.

Protein is a critical macronutrient required for growth, development, and maintenance of physiological functions. Globally, dietary protein sources vary considerably and are shaped by regional agricultural systems, cultural traditions, and food availability [2]. In many high-income regions, including parts of Europe, animal-based foods have historically been dominant protein sources, partly due to livestock-oriented agricultural practices. It was seen that pastureland predominated over arable land, and livestock production was more feasible than crop cultivation [1]. In contrast, in many tropical and low- and middle-income regions, plant-based protein sources such as pulses and cereals have long been central to dietary patterns [2].

Increasing awareness of the environmental and health impacts of current food systems has driven a shift toward plant-rich dietary patterns in many European countries. These plant-rich diets are generally associated with lower environmental footprints and may provide a wide range of nutrients when appropriately balanced [3]. Whole plant foods, including pulses, grains, seeds, and nuts, are not only important protein sources but also supply dietary fiber, essential micronutrients, and bioactive compounds that may contribute to disease prevention. Some of these components have been associated with reduced risk of non-communicable diseases (NCDs), including cardiovascular diseases, type 2 diabetes, and hypertension [3].

A growing body of evidence highlights the central role of gut health in mediating these effects. Dietary fiber and polyphenols present in pulses can modulate gut microbiota composition, enhance microbial diversity, and stimulate the production of short-chain fatty acids (SCFAs), which play key roles in maintaining intestinal barrier integrity and regulating immune and metabolic function [4,5]. Fermentation processes further enhance these benefits by improving the digestibility of proteins within the food matrix, increasing the bioavailability of micronutrients, and enhancing the fermentability of dietary fibers [6].

Although some plant proteins contain lower levels of specific essential amino acids, traditional dietary practices, such as combining pulses with cereals, can provide a balanced amino acid profile [7]. However, challenges remain because pulses contain compounds traditionally referred to as antinutritional factors (ANFs), including phytates, tannins, and protease inhibitors, which reduce nutrient bioavailability by forming insoluble complexes, particularly with divalent minerals [8]. It is worth noting that these ANFs may also lead to positive health effects; increasing evidence suggests that these compounds can also exert beneficial bioactive effects, such as antioxidant, anti-inflammatory, and microbiota-modulating activities, when consumed in appropriate amounts [9]. Processing techniques such as soaking, germination, cooking, and fermentation play a critical role in optimizing this balance by reducing undesirable effects while enhancing functional properties [10]. At the same time, the growing market for plant-based alternatives has led to the development of highly processed products designed to mimic animal-derived foods. While these products may contribute to reduced animal product consumption, concerns have been raised regarding their nutritional quality, degree of processing, and environmental impact [11]. In this context, minimally processed pulses offer a promising alternative, providing nutrient-dense, functional, and sustainable food options. Based on the literature on pulse processing, fermentation was emphasized in this review because, compared with processing methods that primarily act through leaching, heat denaturation, or activation of endogenous seed enzymes, fermentation offers a broader and more tunable biochemical mechanism for optimizing pulse matrices. Through microbial acidification and the activity of enzymes such as phytases, proteases, α-galactosidases, β-glucosidases, esterases, tannases, and cell-wall-degrading enzymes, fermentation can simultaneously reduce several ANFs, while improving mineral bioaccessibility, protein digestibility, and the release of bound phenolics [12,13].

Recent reviews have addressed important but separate aspects of the pulse-gut-metabolic health axis. Zhao et al. summarized broad pulse-related health outcomes, but that scoping review primarily mapped clinical and observational outcomes [14]. Marinangeli et al. reviewed the limited human evidence on the effect of dietary pulses on human gut microbiota; only five human studies were eligible in their analysis [15]. Kadyan et al. focused on the prebiotic potential of specific compounds such as pulse-derived resistant starch for aging-associated gut and metabolic health [16]. Recent RCT-based evidence further suggests that human studies support selected cardiometabolic outcomes but not many microbiome-mediated mechanisms [17]. Reviews of fermented pulses have further detailed how microbial processing can reduce antinutritional factors and enhance bioactive compounds, but these remain primarily processing-focused and do not fully integrate human gut and metabolic health evidence across pulse types [12,13].

Although previous reviews have provided valuable foundations, significant knowledge gaps remain. An integrated synthesis is still needed to clarify how different pulse types, consumed as whole-food matrices or as sources of extracted bioactive compounds, influence gut mucosal integrity, microbiota composition, and metabolic health. Furthermore, the pulse-specific mechanisms through which different pulse types modulate particular bacterial populations and microbial functions remain insufficiently understood. The limited number of RCTs constrains the ability to draw robust conclusions regarding the effects of pulses on gut and metabolic health outcomes. In addition, the comparative effects of fermentation across different pulse types and their implications for gut and metabolic health have not been systematically evaluated.

Therefore, this review synthesizes current evidence on the role of different pulses in modulating gut and metabolic health, while considering their nutritional, whole-food and bioactive matrix effects, microbiota modulation, metabolic outcomes, fermentation, and sustainability implications. This synthesis is particularly timely because pulse consumption remains low in many European countries despite dietary recommendations and sustainability arguments. At the same time, NCDs continue to represent a major global public-health challenge, with diet-related risk factors contributing substantially to disease burden. By integrating evidence across these domains, this review seeks to clarify the potential role of pulses in supporting healthier, more sustainable dietary patterns and facilitating the transition toward sustainable food systems.

## 2. Methods

A narrative review was conducted based on a comprehensive literature search of scientific databases to identify relevant studies on pulses, gut microbiota, metabolic health, and sustainable diets. PubMed, Scopus, MDPI database, and Google Scholar were searched for peer-reviewed articles published between 2015 and 2025, with selected 2026 publications included where relevant during revision. Search terms (used individually and in combination) included ’pulses’, ’legumes’, ’plant-based proteins’, ’gut health’, ’short-chain fatty acids’, ’metabolic health’, ’non-communicable diseases’, ’fermentation’, ’antinutritional factors’, and ’sustainable diets’. The reference lists of key publications and reviews were also manually screened for additional relevant studies.

Inclusion criteria were broad, encompassing original studies (in vitro experiments, in vivo animal models, and human clinical/observational studies) published in English that examined pulse consumption or pulse-derived components in relation to at least one of the following: nutritional composition or bioactive compounds of pulses; gut health outcomes (e.g., microbiota modulation, short-chain fatty acid production, intestinal barrier function); metabolic health or NCD-related outcomes (e.g., blood lipid profile, glycemic control, inflammation); effects of food fermentation on the nutritional or functional properties of pulses. Exclusion criteria ruled out studies focusing on non-edible pulse species or animal feed applications, given their limited translational relevance to human nutrition and dietary recommendations. Non-peer-reviewed sources (e.g., conference abstracts, opinion pieces) and articles not addressing health impacts or processing of pulses were excluded. All included evidence was synthesized qualitatively. Key findings were organized into thematic sections based on pulse types, nutritional composition, gut and metabolic health outcomes, and effects of fermentation. The synthesis emphasizes fundamental findings and highlights their implications for promoting pulses for sustainable and healthy dietary transitions.

## 3. Findings and Discussion

### 3.1. Protein-Rich Plant-Based Foods and Their Global Consumption

**Figure 1:**
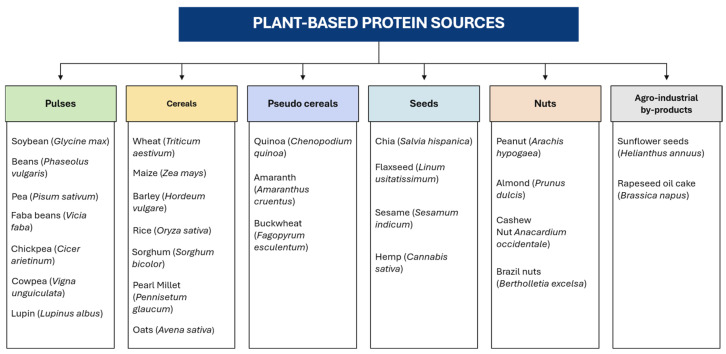
Categorization of plant-based protein sources. The figure represents only the common protein sources in each category, not all.

Plant-based protein sources can be broadly categorized into pulses, cereals, pseudocereals, seeds, nuts, and agro-industrial by-products [18]. Figure 1 shows this classification.

Pulses are the dried edible seeds of leguminous plants (family Fabaceae); commonly consumed pulses include soybeans (*Glycine max*), dry beans (*Phaseolus vulgaris*), chickpeas (*Cicer arietinum*), peas (*Pisum sativum*), faba beans (*Vicia faba*), cowpeas (*Vigna ungulculata*), and lupins (*Lupinus albus*), each providing unique profiles of protein and bioactive compounds [19]. Pulses form a cornerstone of global plant-protein nutrition due to their high protein content (typically 17–30% by dry weight) and substantial contributions of dietary fiber, resistant starch, and micronutrients [20]. Cereals (grass family Poaceae) are generally lower in protein (about 7–15% by dry weight) but remain major protein sources in many diets, especially in low- and middle-income countries. Though cereal proteins are limited in lysine, they synergize with the amino acid profile of pulses, a basis for traditional dietary combinations like maize and beans or rice and lentils, which together achieve a complete protein profile [19].

Pseudocereals such as quinoa (*Chenopodium quinoa*), amaranth (*Amaranthus* spp.), and buckwheat (*Fagopyrum esculentum*) contain intermediate protein levels (~12–18%) with relatively balanced amino acid ratios, including higher lysine content than true cereals [21]. Seeds and nuts (e.g., chia, sunflower, almonds, walnuts) are nutrient-dense sources of protein (often 15–30%), healthy fats, vitamins, and minerals, albeit typically consumed in smaller quantities due to their high caloric density [18]. Agro-industrial by-products (like oilseed cakes from sunflower or rapeseed) can contain very high protein concentrations (up to ~50%), along with fiber and phenolics, making them promising sustainable protein sources if challenges like off-flavors and antinutrients can be addressed [22].

Pulses play an increasingly significant role in global food security and sustainable diets. In 2022, approximately 96 million tons of pulses were produced worldwide, equating to an average per capita daily intake of ~20 g. With rising recognition of their environmental sustainability and health benefits, global pulse production is projected to reach 125 million tons by 2032, potentially increasing per capita consumption to ~25 g/day [23]. However, geographic disparities in consumption persist: Latin America & the Caribbean, Sub-Saharan Africa, and South Asia historically have the highest pulse intakes (often ~35 g/day per person), reflecting the centrality of pulses in traditional diets [24]. In contrast, Europe has relatively low pulse consumption (ranging from 1 to 18 g/day) [25], well below dietary recommendations. European dietary guidance increasingly promotes regular pulse consumption, with several national guidelines recommending daily or frequent intake of pulses [26]. For instance, Spanish dietary guidelines recommend at least 4 servings a week (one serving 50–60 g dry pulses) [27], and the Danish dietary guidelines suggest the intake of 100 g/day [28]. Bridging these gaps will require not only consumer education but also innovation in food processing and product development to enhance the appeal and convenience of pulse-based foods.

### 3.2. Nutritional and Functional Properties of Pulses

**Figure 2:**
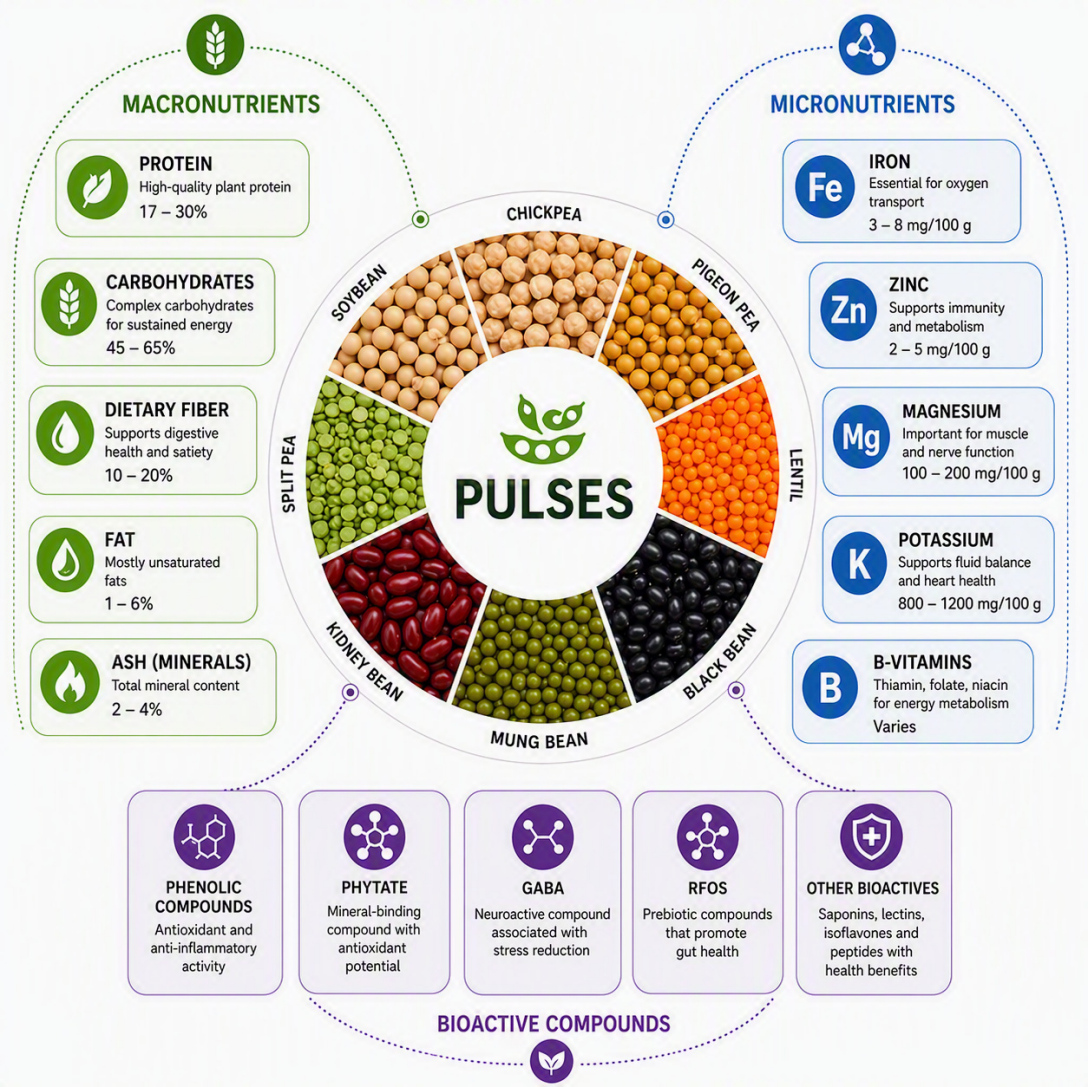
Overview of the major macronutrients, micronutrients, and bioactive compounds present in pulses. Note. Values are approximate ranges (per 100 g).

Figure 2 shows a schematic representation of pulses and their major nutritional components, including macronutrients, micronutrients and bioactive compounds. From a nutritional perspective, pulses are important because they combine plant protein, complex carbohydrates, dietary fiber, minerals and diverse non-nutrient bioactive compounds in a relatively shelf-stable food matrix. Their composition varies by species, cultivar, growing environment and processing, but a typical pattern is high carbohydrate, moderate-to-high protein, low fat and a small mineral-rich ash fraction [29]. Protein is one of the nutritional features of pulses. Whole-seed pulses generally contain about 17–30% protein on a dry basis [30]. Pulse proteins are particularly valuable because they supply lysine, an amino acid that is often limited in cereals, although pulses tend to be lower in sulfur-containing amino acids such as methionine and cysteine; therefore, cereal-pulse combinations can improve overall dietary protein quality [29].

Pulses are excellent sources of dietary fiber (roughly 10–20% of dry weight) with a mixture of insoluble and soluble fractions [30]. Fiber supports gastrointestinal function, satiety and fermentation by gut microbiota, while also contributing to the technological functionality of pulse flours [29]. Carbohydrates usually represent the largest macronutrient fraction (~45–65%), with starch as the major reserve carbohydrate in most pulses, except for lupins, which contain much less starch and relatively more fiber and fat [29]. Pulses are also rich in essential micronutrients such as potassium, zinc, iron, B vitamins, vitamin A, and vitamin E [31].

Collectively, the pulse nutrients and phytochemicals in pulses make them nutritionally dense components of a healthy diet. Beyond basic nutrition, pulses contain multiple bioactive compounds such as polyphenols (e.g., phenolic acids, flavonoids), phytosterols, resistant oligosaccharides, and specialized peptides, which are associated with health-protective effects [32]. Phenolic acids, flavonoids, anthocyanins and tannins contribute to antioxidant capacity, color and sensory properties, and may exert anti-inflammatory or antimicrobial effects depending on bioavailability and food matrix [29,32]. Gamma-aminobutyric acid (GABA) is a non-protein amino acid present in pulses that can increase during germination and fermentation alongside increases in polyphenols and antioxidant activity [12].

Phytate (phytic acid) is the main phosphorus storage compound in seeds and can bind minerals such as iron, zinc and calcium, reducing their bioavailability; however, it may also exert antioxidant or metabolic benefits [8]. Processing methods such as soaking, germination and fermentation can reduce phytate and improve mineral bioavailability [10,33]. Fermentation was also shown to increase polyphenol content in various seed plants [34]. Raffinose-family oligosaccharides (RFOs), including raffinose, stachyose and verbascose, are indigestible in the small intestine and fermented in the colon and may contribute to prebiotic effects; however, they can also cause flatulence and gastrointestinal discomfort at high doses [29]. Other bioactives include saponins, lectins, enzyme inhibitors and isoflavones. Overall, pulses provide a complex matrix where bioactive compounds interact with nutrients, contributing to antioxidant, fermentable and functional properties that complement their nutritional value. The dual role of ANFs, pros and cons, and the impact of processing on their risk–benefit profile are discussed in Section 3.3.

#### 3.2.1. Pulses, Metabolic Health and Non-Communicable Diseases (NCDs)

Diets rich in pulses have been linked to reduced risk of NCDs through multiple mechanisms. Regular pulse consumption has been shown to improve cardiometabolic health indices by helping maintain healthy blood lipid profiles, better glycemic control, and blood pressure regulation [31]. Additionally, pulses’ high antioxidant and anti-inflammatory compounds contribute to lowering oxidative stress and inflammation, and they support a more diverse and balanced gut microbiota composition, all of which are factors in NCD prevention [30]. Table 1 provides an overview of key health benefits observed in seven commonly consumed pulses based on in vitro and in vivo studies, highlighting associated bioactive components with these effects. What follows is a synthesis of these findings, emphasizing the major health effects and underlying bioactives of each pulse type.

Soybeans (*Glycine max*) are uniquely rich in protein (up to ~35%) and contain isoflavones, peptides, and polyphenols. These components contribute to a range of health effects. For example, black soybean diets (20% of a high-fat diet) in mice significantly improved metabolic parameters, reducing weight gain, adiposity, serum cholesterol and glucose levels, and liver fat accumulation, effects attributed to soy’s abundant fiber and polyphenols [35]. In human trials, soy protein and isoflavone supplementation in postmenopausal women improved bone health markers (increasing bone mineral density and reducing menopausal symptoms), although it also modestly influenced thyroid hormone levels, indicating that long-term endocrine effects require careful consideration [36]. Additionally, soy-derived bioactive peptides have demonstrated bioactive potential in preclinical models: a peptide fraction from soybean residue exhibited notable antioxidant activity and selective cytotoxic effects against human cancer cell lines, suggesting a possible protective role against cancer development [37].

Beyond soybeans, other commonly consumed pulses also demonstrate diverse bioactive profiles relevant to cardiometabolic and NCD-related outcomes: Common beans (*Phaseolus vulgaris*), including a variety of types, one of which is pinto beans, with a protein content of 15–25%, have shown cholesterol-lowering, antidiabetic, and antiproliferative effects. In one study, hamsters on a high-saturated-fat diet had significantly reduced total and LDL cholesterol, triglycerides, and hepatic lipid accumulation when their diet was supplemented with either whole pinto beans or hydrolyzed bean protein; the whole bean was more effective, likely due to complementary bioactive compounds in the cotyledon (rich in tannins, saponins, and other phytochemicals) that synergistically seemed to enhance lipid metabolism improvements [38]. Studies investigating phenolic extracts from various common bean cultivars have shown antioxidant and anti-cancer properties: for instance, polyphenol-rich extracts from common beans inhibited the proliferation of colon and breast cancer cells, demonstrating that health effects arise from the combined action of multiple bean phytochemicals rather than any single compound [39]. In diabetic animal models (rats), green bean (immature *P. vulgaris*) extracts significantly lowered blood glucose and oxidative stress markers, enhanced insulin secretion, and inhibited digestive enzymes, effects credited to bioactive constituents such as phytosterols and flavonoids [40].

The common bean rows in Table 1 are particularly relevant to the food-matrix argument because whole pinto beans produced stronger lipid-related effects than hydrolyzed bean protein, suggesting that cotyledon-associated phytochemicals and fibers may contribute synergistically to lipid metabolism. A more detailed discussion of the underlying mechanisms is presented in Section 3.2.2.

**Table 1:** Overview of health benefits from the intervention studies of seven common protein-rich pulses conducted in vitro and in vivo in experimental models and human intervention studies.

Pulses	Treatment	Study Model	Associated Health Effects	Attributed Compounds	References
Soybean	Purified peptide F2-c isolated from alcalase hydrolysate of black soybean protein	In vitro—human liver (HepG2), lung (MCF-7) and cervical (Hela) cancer cell lines.	↑ 95.5% Antioxidant capacity (DPPH) ✓ Inhibits proliferation of three cancer cell lines (HepG2, MCF-7, HeLa) ✓ Anti-tumor activity	Novel tetrapeptide Leu/Ile–Val–Pro–Lys (L/I-VPK)	[37]
	20% soybeans in a HFD for 12 weeks	In vivo—C57BL/6 mice	↓ Cholesterol ↓ Serum glucose ✓ Reduction in hepatic steatosis, adipocyte size	Anthocyanins, isoflavones, dietary fibers in black soybeans	[35]
	15 g soy protein with and without 66 mg isoflavone. Daily for 6 months	In vivo human—RCT with 200 women after their menopause	↓ 13.5% Fasting glucose ↓ 62.6% Insulin resistance (HOMA-IR) ↓ Systolic blood pressure ✓ Reduced menopausal symptoms	Soy isoflavones (Genistein, Daidzein, Glycitein)	[36]
Common beans	Phenolic extracts from common beans at various doses	In vitro—colon cancer cell lines (HT29 and HCT116) and breast cancer cell line (MCF7)	↓ ~34% Intracellular reactive oxygen species (ROS) ✓ Antioxidative ✓ Antiproliferative activity	Total phenolics, flavonoids, and proanthocyanidins	[39]
	White common bean extract, 1.5 g taken 30 min before meals	In vivo human—RCT with 90 subjects with type 2 diabetes	↓ 0.71% HbA1c (glucose metabolism marker) ✓ Improved glucose metabolism	Highly active α-amylase inhibitor	[41]
	High saturated fat diet + 5% whole pinto beans or + 0.5% hydrolyzed pinto beans for four weeks.	In vivo—Male golden Syrian hamsters	↓ up to 53.69% Total cholesterol ↓ up to 89.1% HMGCR reductase (cholesterol synthesis) ✓ Cholesterol lowering	Polyphenols, flavonoids, tannins, saponins and bean cotyledon	[38]
Peas	Soluble proteins extracted from peas germinated for various days	In vitro—cell-based assay and measuring proteinase inhibitory action	↑ 43.1% inhibition of protein denaturation ↑ 34.0% proteinase activity ✓ Anti-inflammatory	Soluble proteins and bioactive peptides	[42]
	Purified pea glycoprotein PGP2 at different doses	In vivo—Male ICR mice, diabetes induced by intraperitoneal injection of streptozotocin (STZ)	↓ 20.7% Glycosylated serum protein (GSP) ↑ 31.5% Insulin ✓ Antidiabetic	Glycoprotein and polysaccharides	[43]
Faba beans	Peptides from digested faba bean flour	In vitro—Caco-2 cells	~15–20% DPP-IV inhibition ✓ Antihypertensive ✓ Antioxidative activity ✓ Antidiabetic	Globulins (legumin, Vicilin, Convicilin), bioactive peptides	[44]
	Methanolic extracts of faba beans (500 mg/kg BW)	In vivo—Male Swiss albino mice, diabetes induced by Alloxan	↓ 42.4% Blood Glucose ↓ 14.9% Total cholesterol ↓ 32.8% Triglycerides ↓ 20.6% LDL ↑ 23.1% HDL ✓ Antidiabetic effect ✓ Antioxidative	Flavones, flavonols, anthocyanins, phenolic acids	[45]
Chickpeas	Chickpea sprout isoflavone extract (10–60 µg/mL)	In vitro—human breast cancer cell lines SKBr3 and MCF-7	↑ Apoptotic cell populations Activation of apoptotic signaling pathways ✓ Anti-tumor activity	Isoflavones from chickpea sprouts (Biochanin, genistein, daidzein	[46]
	Chickpea extract (250 and 500 mg/kgBW)	In vivo—rat hepatotoxicity model using male Wistar albino rats	↓ 45.3% Total cholesterol ↓ 68.9% LDL cholesterol ↓ 29.7% triglycerides ↑ 61.2% HDL ✓ Antilipidemic effect ✓ Hepatoprotective ✓ Anti-inflammatory effect	Phenolic compounds and isoflavones.	[47]
Cowpea	Aqueous and ethanolic extracts of cowpeas (20–120 μg/mL)	In vitro antioxidant and anti-inflammatory assays	↑ 41.4% stronger α-amylase inhibitory activity (ethanol extracts) ↑ 97.1% HRBC membrane stabilization	Phenolic content and bioactive sugars in the cowpea extract	[48]
	Cowpea ethanolic extracts with isoflavone content, 1.25, 2.5, or 5 mg/kg BW	In vivo—female Rattus norvegicus (rats) after ovariectomy—model for postmenopausal estrogen deficiency	↓ ~35% Malondialdehyde (MDA, stress marker) ✓ Reduced oxidative stress ✓ It may prevent bone mineral density loss during menopause	Phytoestrogenic compounds (isoflavone-like) in cowpea extract	[49]
	20% Cowpea incorporated into an HFD for 6 weeks. Various preparations	In vivo—male Wistar rats induced Hypercholesterolemia with HFD	↓ ~25% Total cholesterol ↑ Antioxidative capacity ✓ It may be effective in the treatment of hypercholesterolemia	Soluble dietary fibers and antioxidants in the cowpea	[50]
Lupin	Purified γ-conglutin from lupin seeds (95% purity) was subjected to GID	In vitro—pancreatic β-cells and human skeletal muscle myotubes	↑ 1.4-fold glucose uptake ↑ 2-fold glycogen content. ✓ Antidiabetic activity through the activation of various insulin-signaling pathways	**γ**-Conglutin-derived peptides generated after gastrointestinal digestion	[51]
	Lupin protein hydrosylate (100 mg/kg), intragastrically for 12 days	In vivo—autoimmune encephalomyelitis induced in ApoE^−/−^ male mice	↓ 11.9% cholesterol ↓ 10.1% LDL cholesterol ↓ 15.0% Triglycerides ✓ Increased antioxidant protection ✓ Reduced the risk of atherosclerosis	Lupin protein hydrolysate peptides	[52]
	Three diets: 1. Lupin 25 g/d. Milk protein 25 g/d. 3. Milk protein plus 1.6 g/d arginine. For 28 days	In vivo human—RCT with 72 hypercholesterolemic subjects	↓ 4% total cholesterol ↓ 4% LDL cholesterol ↓ 9% triacylglycerols ✓ Improved cardiovascular risk markers	Lupin seeds, with L-arginine identified as a likely partial contributor	[53]
Mixed pulses	Calorie-restricted diet enriched with mixed pulses (beans, chickpeas) 100 g/meal for 16 weeks	In vivo—human, RCT with 127 subjects with prediabetes	↓ ~7.5% total cholesterol ↓ ~8.5% LDL cholesterol ↓ ~3.5% HbA1c (Hemoglobin A1c) ✓ Improved metabolic markers	Dietary fiber from pulses	[54]

↑ Ascending arrows indicate an increase in values; ↓ Descending arrows indicate a decrease in values; RCT, Randomized Control Trials; DPP-IV, Dipeptidyl Peptidase IV, BW, Body Weight; GID, Gastrointestinal Digestion; HFD, High Fat Diet; LDL, low-density lipoprotein cholesterol; HDL, high-density lipoprotein cholesterol; DPPH, 2,2-Diphenyl-1-picrylhydrazyl Radical Scavenging Assay.

Whereas common beans provide evidence for lipid- and glucose-related effects involving whole-bean matrices and phenolic fractions, peas highlight the contribution of germination-induced proteins, peptides, and glycoproteins to anti-inflammatory and antidiabetic mechanisms. Peas (*Pisum sativum*) contain approximately 16–28% protein; various studies have shown benefits for metabolic and immune health. Pea-derived protein fractions and glycoproteins can exert antidiabetic effects: purified pea seed glycoprotein (PGP2) administration in diabetic mice improved insulin secretion, glycemic control, and organ protection (liver, kidneys and pancreas), consistent with an anti-diabetic role [43]. Further, in vitro experiments reveal that prolonged germination of peas enhances the anti-inflammatory properties of their protein fractions, indicating that certain bioactive peptides become more potent with germination duration [42].

Faba beans (*Vicia faba*) extend this discussion toward peptide- and polyphenol-mediated mechanisms, although the current evidence remains largely preclinical. Faba beans with protein contents around 28% also contain beneficial bioactives, though much evidence currently comes from laboratory studies. In a comparative in vitro investigation, protein-rich faba bean peptides isolated after simulated digestion showed antioxidant activities in Caco-2 cell models, comparable to or exceeding soy and pea peptides under similar conditions [44]. In diabetic mice, administration of a methanolic faba bean extract (500 mg/kg body weight) resulted in improved glycemic control and pronounced antioxidant effects, including upregulation of endogenous antioxidant enzymes (catalase (CAT), glutathione peroxidase (GPx), superoxide dismutase (SOD) and antibacterial activity against gut pathogens [45]. These findings suggest that faba beans harbor polyphenols, peptides, and fibers with anti-diabetic, anti-hypertensive, and antioxidant effects, although more human data are needed to confirm their clinical relevance.

Chickpeas (*Cicer arietinum*) 13–27% protein provide another example in which phenolics, isoflavones, fermentable fibers, and protein fractions may converge on oxidative stress, inflammation, and glycemic regulation. In a rodent model of liver injury, chickpea extracts (250–500 mg/kg) showed hepatoprotective effects comparable to the antioxidant drug silymarin: treated rats exhibited lower inflammation (reduced TNF-α), decreased oxidative stress (lower lipid peroxidation, higher antioxidant enzymes like CAT, SOD, GPx), and improved liver enzyme profiles [47]. In in vitro experiments, chickpea sprout isoflavone extracts at various concentrations significantly slowed the growth of human breast cancer cells via pro-apoptotic mechanisms. These anti-tumor effects were linked to bioactive compounds such as daidzein derivatives (biochanin A and formononetin), saponins, and even microbial metabolites like butyrate that can result from fermentable fibers in chickpeas [46]. Moreover, in a diabetic rat model, adding chickpea flour extracts to a high-fat diet improved glycemic control and reduced inflammatory markers. The authors attributed the effect to the high soluble fiber content acting as a prebiotic and promoting better insulin sensitivity [55].

Cowpeas (*Vigna unguiculata*), containing ~23–28% protein, illustrate how pulse bioactives may interact with hormonal, oxidative-stress, and lipid-metabolism pathways, but most available evidence is reported to provide health benefits especially in the context of metabolic and cardiovascular health. In a mouse model of post-menopausal metabolic decline, cowpea isoflavone-rich extracts (0.5–5 mg/kg) produced cardiovascular protective effects by attenuating atherosclerotic changes and improving bone mineral density during estrogen deficiency (menopause) [49]. In a separate rodent study, diets enriched with cowpeas (raw, boiled, or sprouted forms) for six weeks protected against diet-induced obesity and dyslipidaemia, significantly lowering total and LDL cholesterol while raising beneficial HDL levels; notably, raw (minimally processed) cowpeas retained the highest antioxidant activity, highlighting an inverse relationship between processing intensity and some health benefits [50]. In vitro assays of cowpea extracts also demonstrated anti-diabetic and anti-inflammatory potential, showing α-amylase enzyme inhibition and membrane-stabilizing effects, alongside strong radical-scavenging antioxidant activity [48].

Last but not least, lupins (*Lupinus* spp.) with approximately 27–45% protein emerge as potent functional ingredients due to their rich content of proteins and unique peptides. Clinical evidence indicates that lupin consumption (approximately 25 g per day) can beneficially modulate cardiovascular risk factors: in a controlled trial, diets enriched with lupin protein significantly lowered total and LDL cholesterol, triglycerides, and blood pressure in hypercholesterolemic individuals compared to a milk-protein control diet [53]. In vivo animal studies suggest additional benefits: a lupin protein hydrolysate administered to mice in a model of autoimmunity (experimental autoimmune encephalomyelitis, an MS-like condition) attenuated disease severity, hinting at anti-inflammatory and neuroprotective properties [52]. Furthermore, γ-conglutin, a protein fraction from lupin, has demonstrated insulin-mimetic effects in cell-based assays by enhancing glucose uptake and inhibiting dipeptidyl peptidase-4 (DPP-IV), suggesting a mechanism for anti-diabetic action in vitro assays [51].

The literature shows that a wide array of studies supports the NCD-preventive potential of pulses. Evidence from in vitro, in vivo, and limited human trials indicates that pulses can positively influence lipid and glucose metabolism, reduce oxidative stress, and modulate inflammation and blood pressure. These health effects are likely mediated by synergistic actions of multiple components in pulses, including proteins, dietary fibers, and phytochemicals, rather than any single compound. However, the magnitude and consistency of benefits can vary among different pulses and study designs. While pulses like soy, chickpea, and lupin have demonstrated relatively consistent improvements in lipid profiles, glycemic control, or inflammatory markers across studies, others like faba bean and cowpea currently have mainly preclinical evidence, underlining the need for further human research. Overall, Table 1 shows that human evidence is available for only selected pulse types and outcomes, whereas many mechanistic claims remain based on animal or in vitro models. Table 1 also illustrates an important evidence pattern: many studies reported that NCD-related effects are linked to pulse-specific combinations of fiber, phenolics, peptides, isoflavones, and enzyme inhibitors. This supports a food-matrix interpretation of pulse health effects. The mechanistic roles of key constituents such as dietary fiber and polyphenols, and their contribution to gut and metabolic health are discussed in detail in Section 3.4.

#### 3.2.2. Pulses and Gut Health

Gut health is a multifaceted concept encompassing a balanced gut microbiota composition, effective production of microbial metabolites (especially SCFAs), robust intestinal barrier function (intact mucosal integrity and tight junctions), and proper immune modulation within the gut (tolerance of beneficial microbes and controlled inflammatory responses). These factors jointly support optimal nutrient absorption, metabolic regulation, and defense against pathogens [56]. Many of the cardiometabolic benefits described in Section 3.2.1 may, at least partly, be mediated through gut microbiota modulation and SCFA production, which will be discussed below.

The nutritional and bioactive components of pulses have a profound impact on gut health. Dietary fermentable fibers, resistant starches, oligosaccharides, and bioactive peptides derived from pulses can nourish beneficial commensal bacteria (notably Bifidobacterium and *Lactobacillus* spp.), promote SCFA production (particularly acetate, propionate, and butyrate), and enhance the gut’s barrier function. For example, studies demonstrate that fiber and polyphenol-rich pulses increase SCFA levels, reduce harmful putrefactive metabolites, and improve intestinal epithelial integrity. In one in vivo rat study, soy-rich diets selectively increased beneficial lactic acid-producing bacteria while lowering the levels of indole (a potentially toxic microbial metabolite), indicating a detoxifying and prebiotic effect of soy oligosaccharides and fibers [57]. Another study found that feeding pulses to mice led to higher butyrate concentrations and stronger expression of colonic tight junction proteins. The authors linked these changes to reduced local inflammation and improved mucosal immunity [58]. Furthermore, it was shown that SCFAs, especially butyrate, are key mediators of these benefits: they serve as energy sources for colonocytes, reinforce gut barrier integrity, and exert systemic anti-inflammatory effects via G-protein-coupled receptors and epigenetic mechanisms [59]. Notably, the combined presence of fiber, starch, and polyphenols in pulses yields a synergistic effect on gut microbiota modulation and SCFA production, exceeding the impact of isolated components alone [60].

Moreover, the metabolic effects may be partly explained by SCFA-mediated receptor activation and by the whole-pulse matrix. Unlike isolated protein or fiber extracts, intact protein-polysaccharide structures delay digestive enzyme access [61]. In cooked pulses, cotyledon cell walls and associated protein–polysaccharide/starch structures can encapsulate intracellular nutrients, limiting digestive enzyme access and slowing starch and protein bioaccessibility. This structural encapsulation may increase delivery of fermentable substrates to the distal colon, supporting more sustained SCFA production and downstream signaling. In contrast, milling, fractionation, or extraction can rupture cell walls, increase proximal enzyme access, and alter the timing and site of digestion and gut fermentation. The gut-health outcomes summarized in Table 2 provide pulse-specific examples of this matrix-microbiota relationship: soybean, kidney bean, chickpea, cowpea, and lupin interventions repeatedly involve fermentable fibers, resistant starch, oligosaccharides, phenolics, or peptide fractions associated with SCFA production, beneficial taxa, and barrier-related outcomes.

Acetate, propionate, and butyrate are major products of anaerobic fermentation of non-digestible carbohydrates; they act through transporters and receptors, including GPR41/FFAR3, GPR43/FFAR2, and GPR109A/HCAR2 [62]. GPR43 is preferentially activated by acetate and propionate, whereas GPR41 responds to propionate and butyrate, and GPR109A is activated by butyrate; these receptors are expressed in intestinal cells, immune cells, adipocytes, and colon tissue, allowing SCFAs to connect gut fermentation to systemic physiology [62].

Although these SCFA receptor pathways are biologically well established, evidence linking whole-pulse consumption to FFAR2/FFAR3 and GPR109A-mediated metabolic effects in humans remains limited; therefore, these mechanisms should be interpreted as mechanistic plausibility supported mainly by cell, animal, and selected human biomarker evidence rather than as definitive proof of clinical efficacy. In the white common bean extract trial shown in Table 2, the authors explicitly connect increased carbohydrate delivery to the colon with SCFA-producing bacteria and note that SCFAs can stimulate intestinal L-cell secretion of GLP-1 and PYY, hormones relevant to satiety, insulin secretion, and glucose regulation [41]. Thus, the pulse food matrix provides a mechanistic link between structural characteristics of whole pulses, microbial fermentation, SCFA production, receptor activation, and downstream metabolic benefits.

SCFAs may also improve metabolic health through anti-inflammatory and barrier-protective effects. It was shown that SCFAs reduce pro-inflammatory cytokine signaling, including IL-6, IL-1β, IL-8, and TNF-α, partly through reduced NF-κB activation and histone deacetylase inhibition [62]. Reviews of microbial metabolites emphasize that metabolites produced near the gut epithelium can regulate permeability, immune responses, and tight junction pathways [63]. The kidney bean and chickpea studies summarized in Table 2 exemplify this pathway. In the kidney bean extract, the authors linked the increased butyrate or total SCFA production with reduced inflammation and improved tight-junction or mucosal barrier markers, although these findings remain primarily preclinical, conducted in animal studies [58]. The study of cooked chickpea flour showed increased SCFA production and epithelial/mucosal barrier integrity in colitis models [64]. These findings are mechanistically coherent but should be framed as preclinical support rather than definitive human evidence.

Phenolic compounds in pulses add a second, reciprocal microbiota-mediated mechanism. Pulses contain phenolic acids, flavonoids, anthocyanins, tannins, and isoflavones, often concentrated in seed coats and darker-colored varieties; their bioavailability depends on matrix binding, digestion, and microbial metabolism. Because many polyphenols are poorly absorbed in the small intestine, a large fraction reaches the colon, where microbes catalyze hydrolysis, demethylation, dihydroxylation, reduction, and ring cleavage to generate smaller phenolic acids and other metabolites with greater bioavailability [65]. This microbiota-polyphenol relationship has been shown to be bidirectional: microbes transform pulse phenolics into bioactive metabolites, while phenolics can reshape microbial communities through antimicrobial effects, redox modulation, and cross-feeding interactions [65].

The metabolic effects of pulse phenolics are likely mediated through overlapping anti-inflammatory, antioxidant, and microbial pathways. Polyphenol-derived metabolites have been linked to Nrf2 activation, NF-κB inhibition, AMPK signaling, and improved epithelial integrity in mechanistic literature [65]. Pulse-processing evidence also shows that fermentation can release bound phenolics via microbial β-glucosidases, esterases, xylanases, cellulases, and related enzymes, thereby increasing free phenolics and antioxidant activity in several pulse matrices [66,67]. However, pulse-specific human trials isolating phenolic effects remain limited; most human evidence comes from whole-pulse interventions or common bean extract studies, whereas phenolic biotransformation pathways are better supported by in vitro and animal studies.

Taken together, pulse fiber and phenolics likely act synergistically because they enter the colon together within a structurally complex food matrix. Fiber and resistant starch provide fermentable carbon for saccharolytic bacteria and SCFA formation, while phenolics selectively influence microbial ecology and are themselves converted into lower-molecular-weight metabolites. This combined substrate flow may explain why whole-pulse interventions often improve lipid and glycemic endpoints even when isolated mechanisms are difficult to separate. In human trials, the most defensible conclusion is that pulses improve fasting glucose, HbA1c, LDL cholesterol, and total cholesterol in some contexts, with emerging evidence that these effects are partly mediated by microbiome composition, carbohydrate-active functions, SCFAs, bile acids, and amino-acid metabolites [17,54].

**Table 2:** Evidence of the effect of different pulses on gut health as seen in in vivo and in vitro study models with varied treatment conditions.

Pulses	Treatment/Intervention Conditions	Study Model	Gut Health Effects	Attributed Compound(s)	References
Soybean	High-fat diet with 20% soybeans for 12 weeks	In vivo using C57BL/6 mice	↑ *Bacterioides* and *Proteobacteria*. ↑ SCFA	Polyphenols and dietary fibers	[35]
	Diets with 20% soy protein, whole soybean flour, or soy protein plus 2% soya fiber administered for 7 days	In vivo—Wistar rats (cecal microbiota evaluation)	↓ Indole concentrations (a putrefactive compound) in cecal contents ↑ Lactic acid production ✓ Microbiota shift favoring *Prevotella* and other Gram-negative anaerobes	Soybean oligosaccharides and fibers	[57]
Kidney Beans	Whole kidney bean and bean hull (3.5%) and cellulose, for 4 weeks	In vivo—Fischer rats	↑ SCFA production in cecum	Dietary fibers and resistant starch	[68]
	White common bean extract, 1.5 g taken 30 min before meals	In vivo human—RCT with 90 subjects with type 2 diabetes	↑ *Bifidobacterium*, ↑ *Faecalibacterium*, ↑ *Anaerostipes*; ↓ *Weissella*, ↓ *Klebsiella*, ↓ *Cronobacter* ↓ ✓ Microbiota shift	Activated α-Amylase inhibitor	[41]
	Kidney bean extract containing phaseolin, added into an HFD	In vivo—C57BL/6 mice	↑ Butyrate levels in serum and feces ↓ Colonic inflammation ✓ Restored gut barrier integrity (tight junction protein expression)	Phaseolamin (α-amylase inhibitor) contained in the bean extract	[58]
Peas	Low-phytate pea varieties, control pea variety and a no-pea diet for 6 weeks	In vivo—*Gallus gallus* model	↑ Fe bioavailability in low phytate peas ✓ Promoted SCFA-producing bacteria ✓ Improved the functionality of brush border membrane	Reduced phytate, dietary fibers and protein	[69]
	Peas extract, albumin fraction (15 g/kg/day), administered orally for around 3 weeks	In vivo—colitis-induced C57BL/6J mice	↓ Inflammatory markers, cytokines ✓ Improved intestinal barrier properties by increasing the expression of tight junction proteins	Pea albumin	[70]
Faba beans	Soybean meal, partially replaced with high- or low-tannin faba beans 100, 200, or 300 g/kg diet for 5 weeks	In vivo—Turkey birds	↓ *Salmonella* bacteria in cecum ↑ Promoted SCFA production in cecum ✓ Improved mucosal thickness	Reduced tannins and non-starch polysaccharides	[71]
	Commercial feed supplemented with four different faba bean fractions for over 100 days	In vivo—Grass carp	✓ Improved intestinal antioxidative capacity ✓ Improved intestinal villi microstructure	Glucosides, bioactive peptides, polyphenols	[72]
Chickpeas	A diet containing 20% cooked chickpea flour for 3 weeks	In vivo—colitis-induced C57BL/6J mice	↑ Colonic epithelial and mucosal barrier integrity ↑ SCFA production in cecum ✓ Improved microbiota	Dietary fibers and phenolic compounds	[64]
	Chickpea-derived protein and peptide fractions for at least 3 months	In vitro—human fecal samples	↑ Abundances of *Bifidobacterium* and *Weissella* ↑ SCFA production	Antioxidant-rich chickpea albumin peptides	[73]
Cowpea	Zinc biofortified cowpea varieties (50 mg/mL)	In vivo—Red Junglefowl	↑ Villi, goblet cell numbers ↓ Inflammation ✓ Improved intestinal morphology, brush border membrane functionality ✓ Improved gut microbiota	Zinc content, soluble dietary fibers and phenolics	[74]
	Cooked cowpea vs. potato and beef	In vitro—human fecal sample	↑ Production of SCFA ↑ *Bifidobacterium* and *Lactobacillus* abundance ✓ Prebiotic effect	Dietary fibers and polyphenols	[75]
Lupin	Raw or micronized lupin seeds (150–300 g/kg) for 21 days	In vivo—weaned piglets	↑ Myenteron thickness in the distal colon ↑ Production of SCFAs with high lupin diets	Dietary fibers and resistant starch	[76]
	Powdered lupin seeds and broad beans	In vitro—human fecal sample	↑ *Bifidobacterium* spp. ↑ SCFA production	Oligosaccharides, non-starch polysaccharides	[77]
Mixed pulses	Calorie-restricted diet enriched with mixed pulses (beans, chickpeas) 100 g/meal for 16 weeks	In vivo—human, RCT with 127 subjects with prediabetes	↑ *Bifidobacterium*, *Eubacterium rectale*, *Roseburia* spp. ↓ *Bilophila wadsworthia*, *Ruminococcus gnavus* ✓ Improved fiber fermentation	Dietary fiber from pulses	[54]

↑ Ascending arrows indicate an increase in values; ↓ Descending arrows indicate a decrease in values; RCT, Randomized Control Trials; SCFA, Short Chain Fatty Acids; HFD, High Fat Diet.

The following synthesis builds on the findings presented in Table 2, highlighting the pulse components most consistently associated with SCFA production, shifts in gut microbiota composition, and improvements in barrier-related outcomes across in vitro and in vivo studies. Among the different pulse species, soybean provides some of the strongest evidence for modulation of gut microbiota composition. In high-fat diet animal models, soy supplementation not only elevated fecal SCFA levels but can also restore a healthier microbial balance, normalizing an obesity-induced excess of Firmicutes relative to Bacteroidetes [35]. By delivering fermentable oligosaccharides and fiber, soy diets increase lactic acid bacteria and reduce production of protein fermentation by-products like indole, demonstrating a dual prebiotic effect and a decrease in potentially harmful metabolites in the colon [57]. Common beans similarly showed to enhance cecal SCFA output when incorporated into rodent diets, with both whole and de-hulled bean fractions promoting microbial fermentation of fiber and resistant starch [68]. Kidney bean extracts rich in the α-amylase inhibitor phaseolamin improved gut health markers in mice by increasing butyrate levels (in both cecal contents and systemic circulation), preserving gut barrier proteins, and reducing colonic inflammation, partly via activation of PPAR (Peroxisome Proliferator-Activated Receptor) signaling pathways [58].

Pea varieties, especially biofortified low-phytate types, demonstrated additional mineral-focused benefits alongside gut modulation. Such varieties not only promoted SCFA-producing bacteria in poultry and improve cecal fermentation, but also enhanced mineral bioavailability (e.g., iron uptake) and intestinal brush-border enzyme activity, a dual advantage likely due to reduced phytate content combined with fibers and proteins that stimulate beneficial microbes [69]. In mice, pea albumin proteins (notably enriched in protease inhibitors like Bowman–Birk inhibitors) improved colonic barrier function, elevating tight junction protein expression and diminishing mucosal cytokine levels in a colitis model [70]. Faba bean interventions have demonstrated noteworthy gut effects in animal studies: adding low-tannin faba bean to poultry diets increased cecal SCFA production, reduced cecal pH, and even decreased Salmonella colonization, likely because additional fermentable fibers and reduced tannin content fostered a more acidic, inhospitable environment for pathogens [71]. In fish, dietary faba bean extracts improved intestinal morphology and lowered markers of oxidative stress and inflammation without affecting growth performance, underscoring gut benefits beyond growth outcomes [72].

Chickpeas have shown prebiotic and anti-inflammatory effects on the gut; in a mouse-induced colitis model, diets including cooked chickpea flour significantly strengthened the colonic epithelial barrier (increasing mucin and tight junction proteins) and elevated SCFA levels, while mitigating gut inflammation [64]. In in vitro fermentations using human stools, chickpea protein and peptide fractions acted as prebiotics, augmenting the growth of health-associated genera like *Bifidobacterium* and *Weissella*, and boosting SCFA production [73]. Cowpeas, especially when biofortified with zinc, have been shown to improve gut structure and immune status in poultry by increasing villus length and goblet cell numbers and by reducing pathogenic bacteria like *Clostridium*, an effect ascribed to their combined high zinc content and fermentable substrates [74]. Moreover, in human gut fermentation models, cooked cowpea was shown to act as a potent prebiotic, significantly increasing SCFA levels and beneficial bifidobacteria and lactobacilli to a greater extent than a typical carbohydrate-rich meal (potato with beef) [75]. Lupins also display prebiotic properties: diets incorporating raw or micronized lupin seeds in piglets increased colonic SCFA production and improved gut muscular layer thickness, reflecting enhanced fermentation of lupin’s fibers and resistant starches [76]. Consistent with these in vivo results, an in vitro human fecal fermentation study showed that lupin and broad bean flour stimulated *Bifidobacterium* growth and SCFA generation, highlighting the fermentability of their oligosaccharides and non-starch polysaccharides [77].

Notably, several pulse species discussed in the effects on metabolic health Section 3.2.1, including soybean, chickpea, and lupin, also demonstrated consistent effects on gut microbiota composition and function. Across studies, these pulses promoted microbial taxa associated with health benefits, increased SCFA production, microbial modulation, and supported intestinal barrier integrity. These gut-mediated effects may partially explain the improvements in metabolic outcomes reported in Section 3.2.1, including enhanced glycemic control, reduced inflammation, improved lipid metabolism, and protection against cardiometabolic risk factors. Collectively, the evidence suggests that modulation of the gut microbiota represents a common mechanism linking pulse consumption to broader health benefits. However, individual pulse species differ in the mechanisms of action depending on available nutrients and compounds capable of modulating the gut microbiota and gut metabolites, as discussed throughout this section. Furthermore, much of the current evidence is from animal or in vitro models, and human data are limited for certain pulses (notably faba bean and cowpea). These findings highlight the importance of dietary diversity (consuming various pulses) and suggest that optimizing processing methods could further potentiate the gut health benefits of pulses. This processing dimension is addressed in Section 3.3, where fermentation is considered as a strategy to modify both ANF profiles and gut-relevant bioactive compounds.

### 3.3. Fermentation as a Strategy to Improve the Nutritional and Health Effects of Pulses

Pulses naturally contain various secondary plant compounds that, in large quantities, can reduce nutrient bioavailability or cause adverse effects. Traditionally labelled antinutritional factors (ANFs), these include enzyme inhibitors (trypsin, chymotrypsin, and α-amylase inhibitors), lectins, phytates, tannins, oxalates, saponins, and certain indigestible oligosaccharides (notably raffinose family oligosaccharides, RFOs) [8]. The concept of “functional ANFs” is relatively recent and reflects a shift from viewing these compounds solely as nutritional liabilities toward recognizing that, when present at appropriate levels or modified through processing, some ANFs may also contribute beneficial physiological, microbiota-related, and metabolic effects.

Although several pulse-derived ANFs may have functional properties at low or moderate exposure levels, their effects should be interpreted as dose-, matrix-, processing-, and host-dependent rather than universally beneficial. High levels of phytate and tannins can reduce mineral bioavailability through complex formation with iron, zinc, calcium and other divalent minerals, while protease inhibitors and lectins may reduce protein digestibility or cause gastrointestinal effects if pulses are insufficiently processed [8,78]. This is particularly relevant for nutritionally vulnerable groups, including individuals with anemia or low iron stores, pregnant women with increased iron requirements, and older adults, in whom micronutrient inadequacy, inflammation, or reduced dietary intake may increase susceptibility to adverse effects [79]. A universal “safe threshold” for all ANFs is not currently scientifically justified. For phytate, risk is commonly interpreted using diet- or food-level phytate:mineral molar ratios rather than a single toxicological threshold. Suggested desirable ratios include phytate:iron < 1, phytate:zinc < 15, and phytate:calcium < 0.17, although these cut-offs should be interpreted as bioavailability indicators rather than universal safety limits [80]. Thus, “safe levels” of ANFs are best framed operationally as levels at which residual activity no longer compromises nutritional adequacy or causes adverse effects under realistic consumption patterns. The potential metabolic effects of residual ANFs should be discussed together with processing efficacy to balance ANFs, nutritional adequacy and host vulnerability.

There is growing recognition of the beneficial roles of AFNs when present in moderate amounts or after proper processing. Traditional processing techniques (soaking, dehulling, sprouting, cooking, fermentation) have been shown to effectively reduce ANF levels in pulses, mitigating their negative impacts on nutrient absorption. At the same time, appropriate processing can optimize ANF content to harness their benefits rather than eliminate them entirely. Thus, when pulses are properly processed, these so-called functional ANFs can act as useful bioactive compounds that contribute to gut health (by modulating microbial communities and reducing oxidative stress) instead of merely hindering nutrition [9].

Compared with other processing methods, as noted in the Introduction, fermentation offers a unique opportunity to optimize pulse quality by reducing ANFs while simultaneously increasing the bioavailability of nutrients and bioactive compounds. Fermentation is a traditional food processing technique that uses microorganisms to break down macromolecules, typically converting carbohydrates into organic acids and other metabolites. In doing so, fermentation can enhance the nutritional and functional properties of foods. For pulses, fermentation has multiple benefits: it partially pre-digests proteins, improving their digestibility; it increases the bioavailability of minerals and phytochemicals (e.g., by releasing bound polyphenols); it reduces antinutritional factors (like phytate, tannins, and protease inhibitors); and it generates or concentrates novel bioactive compounds (such as antioxidant and anti-inflammatory metabolites) [6]. Table 3 summarizes key findings from studies on various pulses (soybean, common bean, pea, faba bean, chickpea, cowpea, lupin) under different fermentation conditions, including solid-state (SSF) and liquid-state fermentation (LSF) and the resultant changes in nutritional properties and health-related outcomes.

**Table 3:** Effects of pulse fermentation on biological and health-related outcomes evaluated in in vitro and in vivo studies.

Pulses	Fermentation Condition	Study Model	Changes in the Food Matrix After Fermentation and Reported Health Effects (If Any)	References
Soybeans	SSF on soybean meal with *Bacillus* sp. Anaerobic conditions at 37 °C for 24 h.	In vitro *GID*	↓ 86.0% glycinin ↓ 70.3% β-conglycinin ↓ 90.01% trypsin inhibitors decreased by ↑ 8.7% protein digestibility	[81]
	SSF of soybeans with *L. plantarum*, *B. subtilis* and *S. cerevisiae*. Anaerobic conditions at 37 °C for 48 h.	In vivo—Piglets	Higher IgA, IgG and IgM levels and greater villus height in small intestine achieved with fermented soybeans	[82]
Common beans	LSF of red beans using 2% LAB culture at 42 °C for 4 days.	In vitro *GID*	↓ Phytates, trypsin inhibitor activity, saponins, tannins and raffinose oligosaccharides. ↑ 19% Protein digestibility increase	[83]
	Pre-fermentation done by LSF of kidney bean broth with 0.2% yeast at 32 °C for 24 h. Later, 1.5% *L. plantarum* and *L. acidophilus* (1:1) were added for further 24 h.	In vivo—Rat model in HFD	↑ Beneficial gut microbiota, increasing *Lactobacillus* and *Bifidobacterium* while reducing Firmicutes. Improved serum lipid profiles	[83]
Pea	LSF of pea flour with *L. plantarum* (0.5%). Aerobic conditions at 37 °C for 72 h.	In vitro *GID*	↑ 10% Protein digestibility increased ↑ starch digestibility increased ↓ raffinose content ↑ mineral bioaccessibility Mg, Mn, Fe, Cu, Zn	[84]
	LSF of peas with *L. plantarum* Z45 and *L. paracasei Z15* (1 × 10^6^ CFU/mL) at 37 °C for 6 h.	In vivo—Balb/c mice with ulcerative colitis	↑ SCFA (acetate, propionate, butyrate) ↓ colonic inflammation Improved mucosal integrity and restored healthy gut bacterial balance	[4]
Faba bean	LSF of faba beans with *L. delbrueckii* subsp. *bulgaricus* and *S. thermophilus* (1.25%) at 30 °C for 16–20 h.	In vitro *GID*	↑ 32% protein digestibility	[85]
	LSF of faba beans with *L. plantarum* at 30–32 °C for 36 h.	In vivo—Turkey birds	↓ Reduced ammonia production in the cecum ↑ SCFA production	[86]
Chickpea	SSF of chickpeas with *P. pentosaceus* LFSBB12 and *P. pentosaceus* LFSBB13 (5%) at 37 °C for 24–48 h.	In vitro *GID*	↓ 47% Phytic acid ↑ 200% TPC and TFC ↑ 12% protein digestibility	[87]
	LSF of chickpeas with 1% *L. acidophilus* ATCC 4356 and *L. plantarum* subsp. at 37 °C, for 24 h	In vivo-diabetes-induced C57BL/6J mice	↑ 50% abundance of beneficial bacteria like *Akkermansia muciniphila* Improved the Firmicutes/Bacteroidota ratio. ↑ SCFA in caecum Up to 80% α-Glucosidase inhibition Antidiabetic effect	[88]
Cowpea	LSF of cowpeas with *L. plantarum*, at 37 °C for 24–48 h.	In vitro *GID*	↑ 20% Phenolic content 20% ↓ 30% Phytic acid ↑ 15% Protein digestibility	[31]
	Spontaneous fermentation of cowpeas at 37 °C for 48 h.	In vivo—Wistar albino rats	↑ Digestibility of protein and amino acids ↑ Phosphorus bioavailability	[89]
Lupin	SSF of lupin with *L. sakei* at 30 °C, with *P. acidilactici* at 32 °C and with *P. pentosaceus* at 35 °C for 24 h	In vitro *GID*	↑ 15% Protein digestibility	[90]
	SSF of lupin with an activated culture of *B. animalis* subsp. *Lc.*, *longum* subsp. and *B. breve*) At 37 °C for 24 h	In vitro—Caco 2 cells	↓ up to 82% oligosaccharides ↓ up to 67% phytic acid ↑ protein content by around 14% ↑ up to 79% cytotoxicity of cancer cells ✓ anticancer activity	[90]
	SSF of lupin with *Bifidobacterium* spp. at 37 °C, 72 h	In vitro—Caco-2 cells	↑ 50% TPC and TAC-DPPH ↑ 25% TAC-ABTS α-glucosidase inhibition increased, greater cytotoxicity activity	[91]

↑ Ascending arrows indicate an increase in values; ↓ Descending arrows indicate a decrease in values; SSF, Solid State Fermentation; LSF, Liquid State Fermentation; *GID*, Gastro Intestinal Digestion; LAB, Lactic Acid Bacteria; TPC, Total Polyphenol Content; TFC, Total Flavonoid Content; TAC, Total Antioxidant Capacity; DPPH, 2,2-Diphenyl-1-picrylhydrazyl Radical Scavenging Assay; ABTS, 2,2′-Azino-bis (3-ethylbenzothiazoline-6-sulfonic acid) Radical Cation Decolorization Assay; SCFA, Short Chain Fatty Acids.

A key message from Table 3 is that fermentation outcomes differ by pulse substrate, starter organism, fermentation mode, temperature, and duration; therefore, improvements in digestibility, ANF reduction, phenolic release, or SCFA-related effects should be interpreted as process-specific rather than as generic effects of fermentation. While most studies presented in Table 3 showed improvements in nutritional properties, evidence regarding health effects remains limited and is largely confined to a few in vivo animal studies. In one animal study, piglets fed a diet containing ~10% fermented soy experienced greater small intestinal villus growth and increased immunoglobulin levels compared to those fed raw soy, indicating enhanced gut health and immune function [82]. Fermented chickpea products have demonstrated prebiotic and anti-inflammatory effects; in diabetic rodents, a fermented chickpea “milk” (with *Lactobacillus plantarum* and *L. acidophilus*) increased cecal SCFA concentrations and boosted beneficial microbiota populations (*Akkermansia muciniphila*), improving metabolic parameters [88]. Similarly, supplementing the diet of colitic mice with a fermented pea-based yogurt significantly reduced colonic inflammation, preserved mucosal integrity, and restored a healthier microbial balance, effectively acting as a symbiotic therapeutic [4]. These studies presented in Table 3 connect fermentation-induced matrix changes to the SCFA and barrier pathways described in Section 3.2.2., but the evidence remains largely animal-based.

As shown in selected studies in Table 3, fermentation was also associated with beneficial effects on metabolic health. A daily dose of fermented kidney bean broth (produced via sequential yeast and lactic acid fermentation) reduced visceral fat accumulation and improved serum lipid profiles in hyperlipidemic rats, while enriching gut microbiota with *Lactobacillus* and *Bifidobacterium* and decreasing the relative abundance of Firmicutes (often linked to obesity) [83]. In another study, replacing a portion of soy-based feed with fermented faba bean in turkey diets led to higher cecal butyrate levels and lower ammonia, a sign of healthier protein fermentation in the gut microbiota; these changes occurred without compromising animal growth performance [86].

Overall, these studies indicate that fermentation often improves the nutritional profile and health-promoting properties of pulses. Fermented pulses tend to have lower levels of antinutrients and higher levels of beneficial compounds (e.g., increased total polyphenols, isoflavones, and in vitro antioxidant capacities in fermented chickpea products) [87]. In many cases, fermentation also increases gut-health benefits by boosting SCFA production and enriching probiotic bacteria, as demonstrated across few pulses. Importantly, the magnitude and type of improvements depend on the specific fermentation conditions as shown in Table 3 (microbial strains, fermentation method, duration, etc.) and the pulse variety itself, suggesting that tailored fermentation strategies may be necessary to maximize each pulse’s health potential.

Furthermore, future research on pulse fermentation should evaluate the double-edged effect of ANFs using integrated risk-benefit designs. Such studies should quantify both total ANFs content and biologically active fractions before and after processing, distinguish, for example, phytate species and lectin/trypsin inhibitor activity, and relate these measures to dose–response outcomes in humans. Beneficial endpoints should include gut microbiota composition, SCFA, glycemic and lipid responses, and inflammatory markers, while safety endpoints should include iron and zinc status biomarkers, hemoglobin, ferritin, transferrin receptor, plasma zinc, protein digestibility, gastrointestinal symptoms, and adverse events. Studies should also include nutritionally vulnerable populations, or at minimum stratify by baseline iron/zinc status, pregnancy/lactation, age, medication use, and habitual dietary patterns. This approach would help to identify a practical ‘risk-benefit window’ in which processing preserves potentially useful bioactive properties while minimizing risks to mineral bioavailability and nutritional adequacy.

The heterogeneity shown in Table 3 also provides the rationale for standardization and quality control, which are central for translating fermented pulse products from experimental systems to reproducible foods. As shown in Table 3, fermentation studies use different pulse substrates and fermentation conditions; these variables strongly influence the enzymes, metabolites, and biological activity generated in the final product. Therefore, improvements in bioactive compounds or reduction in ANFs should not be interpreted as inherent properties of ‘fermentation’ alone, but rather as outcomes of defined strain, substrate and process combinations [6,12]. Future studies should therefore report detailed information on strain identity, inoculum level, substrate pretreatment, fermentation conditions along with the obtained outputs. Quality control is essential in fermentation, as the process may generate undesirable metabolites when raw materials, starter cultures, or processing conditions are poorly controlled or unhygienic. For example, biogenic amines, including histamine, tyramine, putrescine, and cadaverine, can arise from microbial decarboxylation of amino acids and have been reported in several fermented foods, including some fermented soybean products [92,93]. Although EFSA identified histamine and tyramine as the most food-safety-relevant biogenic amines in fermented foods, regulatory limits remain incomplete, with most formal limits focused on histamine in fish products rather than fermented plant foods [92].

Thus, fermented pulse products intended for wider adoption should be produced under documented process controls, using good/-quality raw materials, hygienic handling, and non-pathogenic starter cultures with low or absent biogenic-amine-forming potential. Overall, fermentation should be viewed as a promising but condition-dependent strategy: its benefits for nutritional quality, gut and metabolic health could be maximized when strains and processing conditions are standardized, reproducible, and verified for safety.

### 3.4. Pulses and Their Role in the Transition Toward Healthy and Sustainable Diets

Pulses are uniquely positioned at the intersection of human health and environmental sustainability. As nutrient-dense foods rich in protein, fiber, and micronutrients, pulses can contribute to healthier diets by lowering the risk of NCDs. Simultaneously, pulses have a much smaller environmental footprint than equivalent animal protein sources. Life cycle assessments (LCA) indicate that per unit of protein produced, pulses emit roughly one-tenth the greenhouse gases (GHG) of beef [94] and require significantly less water (approximately 10-fold less than beef, and about half that of poultry or pork) [95]. These resource efficiencies make pulses a cornerstone of sustainable diets, aligning with global calls to reduce food-related GHG emissions and conserve water resources.

The dual benefits of pulses, health promotion and environmental sustainability, mean that increasing pulse consumption can yield synergistic gains. Diet patterns higher in pulses tend to have better nutrient density (more fiber, folate, iron, etc.) and simultaneously lower environmental impact scores. For example, a recent UK population study found that individuals with higher pulse intakes had superior nutrient intakes and a more favorable EAT-Lancet diet index (reflecting lower GHG and land use) compared to those consuming fewer pulses [96,97]. This is consistent with broader modeling studies: replacing 50% of animal-based protein with plant proteins (including pulses) globally could reduce diet-related GHG emissions by ~30% [98]. These findings underscore pulses’ value as potential foods that advance both nutrition and climate goals. As discussed above, fermentation may further enhance the role of pulses in sustainable diets. It is increasingly recognized as a sustainable food-processing strategy because it can enhance nutrient bioavailability, improve digestibility and sensory quality, reduce antinutritional factors, and extend shelf life while requiring relatively mild processing conditions and fewer inputs than many conventional food-processing technologies [12].

The reviewed papers highlight the multifaceted role of pulses as nutrient-dense, bioactive-rich foods with significant potential to support metabolic health, gut microbiota modulation, and the transition toward sustainable dietary patterns. A central finding of this review is the synergistic interaction between pulse components and the gut microbiota. Fermentable substrates in pulses promote the production of SCFAs, which play a critical role in maintaining intestinal integrity and regulating systemic metabolic processes. While several pulses, such as soybean, chickpea, and lupin, demonstrate relatively consistent benefits across both preclinical and human studies, others, including faba bean and cowpea, remain less extensively studied especially in human populations. This variability highlights the importance of considering pulse-specific bioactive profiles, as differences in fiber composition, polyphenol content, and protein fractions may lead to distinct physiological responses.

Evidence from the studies summarized in this review should be interpreted according to study design and strength of evidence reported. In vitro studies provide useful mechanistic evidence that pulse fibers, resistant starch, oligosaccharides, peptides, and phenolics can influence microbial fermentation, SCFA production and antioxidant activity. However, these models do not fully capture host absorption, immune regulation, background diet, inter-individual microbiota variability, or long-term adaptation. Animal studies provide stronger biological plausibility by demonstrating effects on SCFA production, gut barrier markers, inflammation, lipid metabolism, and glycemic control in controlled settings, but species-specific microbiota, dosing levels, and disease models limit direct extrapolation to humans. Therefore, these findings should be interpreted as preclinical support rather than definitive evidence of clinical efficacy.

Human evidence is more directly relevant but remains comparatively limited and heterogeneous. Human RCT support is strongest for selected cardiometabolic outcomes, pulse interventions have been shown to improve total cholesterol, LDL-cholesterol, and fasting glucose in some contexts, although effects are not consistent across all outcomes and may depend on pulse type, dose, food format, trial duration, population, and background diet [17,31]. However, currently limited human RCT support exists for microbiome-mediated mechanisms, gut barrier function, inflammatory pathways, and fermented pulse products specifically. For gut microbiota outcomes, the direction of evidence is generally consistent with preclinical findings, including greater fiber fermentation, enrichment of selected beneficial taxa, and changes in SCFA-, bile acid-, or amino-acid-related metabolites in some studies. Nevertheless, human findings remain inconsistent because trials differ in sequencing methods, endpoints, intervention foods, dose, duration, and participant characteristics [17]. Future studies should prioritize long-term, pulse-specific human RCTs with standardized doses, with clearly described processing conditions, and integrated microbiome, metabolomic, inflammatory, and gut-barrier outcomes.

Fermentation appeared particularly relevant for the scope of this review because it integrates several mechanisms within a single process. In addition to reducing phytate, tannins, protease inhibitors, and RFOs, fermentation can promote protein pre-digestion, mineral decomplexation, phenolic release, bioactive peptide formation, and gut-microbiota-relevant metabolite production. This multi-target effect is especially important in pulses, where the same compounds historically described as ANFs may also contribute antioxidant, anti-inflammatory, or microbiota-modulating effects when present in appropriate amounts or transformed by processing. However, most studies on processing technologies remain at experimental or pilot scale, and there is a need to translate these findings into scalable, consumer-acceptable food systems.

Beyond health outcomes, pulses play a critical role in the broader context of sustainable food systems. Their relatively low environmental footprint, combined with high nutritional value, positions them as key components in strategies aimed at reducing the environmental impact of diets while improving public health. However, despite these advantages, consumption remains suboptimal in many regions, particularly in the Nordic countries. In Sweden, pulse consumption contributes to only 2% of consumers’ daily energy intake [99]. According to the latest Danish National Survey of Diet and Physical Activity (DANSDA 2021–2024), only 0.8% of the Danish population meets the national recommendation for pulse consumption [100]. These intakes are also well below national and international dietary recommendations (often 50–100 g/day) [20,101]. This persistent gap between recommendations and actual intake highlights the need for coordinated efforts across policy, industry, research, and the public-health sector, which are discussed in future outlook in Section 4.

Barriers such as cultural preferences, limited product diversity, and the growing reliance on ultra-processed plant-based alternatives may hinder wider pulse adoption. While minimally processed pulses offer substantial health benefits, the increasing demand for convenience has driven the development of highly processed plant-based products. These products do not always retain the nutritional advantages of whole pulses and may introduce additional concerns related to additives and processing intensity. Therefore, future efforts should focus on developing pulse-based foods that are both nutritionally optimized and aligned with consumer preferences.

The work presented here is inherently limited by its non-systematic design and reliance on heterogeneous studies differing in pulse type, processing method, study design, and outcome assessment, which may introduce selection bias and limit direct comparability across findings. Looking forward, three key priorities emerge. First, there is a need for well-designed, long-term human intervention studies to strengthen the evidence base and clarify dose–response relationships. In parallel, the application of multi-omics approaches, including transcriptomics, metabolomics, and microbiome analyses, could provide deeper mechanistic insights into how pulses and their bioactive components influence host physiology and gut microbial function. Second, advances in fermentation technologies should focus on scalable, standardized and sustainable approaches that preserve or enhance nutritional quality. Third, policy and dietary frameworks must support the integration of pulses as central components of healthy and sustainable diets.

## 4. Conclusions

The evidence synthesized in this review demonstrates that pulses are not only protein-dense foods but also rich sources of bioactive compounds that contribute to improved gut health and metabolic outcomes. Through their high content of dietary fiber, plant protein, and phytochemicals, pulses support key physiological processes, including lipid and glucose regulation, modulation of inflammation, and maintenance of gut microbiota balance. The traditional method of fermentation emerges as both an opportunity and a challenge in maximizing these benefits, as it may enhance protein digestibility and reduce so-called ANFs, striking a balance between enhancing bioactivity and preserving sustainability. Pulses offer a practical and effective pathway toward improving both human and planetary health, combining nutritional value with a substantially lower carbon footprint than animal-based protein sources. Their integration into daily dietary patterns should be promoted not only as an alternative to animal-based proteins but as a cornerstone of resilient, health-promoting, and sustainable food systems.

Future outlook and action suggestions to close the gap between dietary recommendations and actual intake, particularly in Nordic countries where pulse consumption remains very low. For policy makers, pulses should be explicitly incorporated into school meals, institutional food-service standards, and public procurement criteria, with targets for regular pulse-based dishes that are nutritionally balanced, culturally acceptable, and aligned with national and Nordic dietary guidance. For industry, product development should prioritize convenient, affordable, and sensory-optimized pulse foods, including ready-to-eat, instant, and controlled-fermentation products that reduce preparation barriers, off-flavors, and digestive discomfort while preserving the nutritional advantages of whole-pulse matrices. For researchers, priority should be given to long-term, adequately designed human RCTs testing defined pulse types, doses, and processing methods, combined with multi-omics approaches, including microbiome and metabolomic profiling, as well as inflammatory, gut-barrier, cardiometabolic, and consumer-acceptability outcomes. For public-health stakeholders and consumers, practical communication should emphasize culinary skills, familiar recipes, gradual replacement of meat with pulses, and culturally adapted meals that make pulses convenient and acceptable in everyday diets. Together, these actions could help translate the health, gut-microbiota, and sustainability potential of pulses into measurable population-level dietary change.

## Data Availability

No new data were created or analyzed in this study. Data sharing is not applicable to this article.

## Abbreviations

The following abbreviations are used in this manuscript:

ABTS	2,2′-Azino-bis(3-ethylbenzothiazoline-6-sulfonic acid) Radical Cation Decolorization Assay
AMPK	AMP-Activated Protein Kinase
ANFs	Antinutritional Factors
BW	Body Weight
CAT	Catalase
DPP-IV	Dipeptidyl Peptidase IV
DPPH	2,2-Diphenyl-1-picrylhydrazyl Radical Scavenging Assay
FFAR2	Free Fatty Acid Receptor 2
FFAR3	Free Fatty Acid Receptor 3
FRAP	Ferric Reducing Antioxidant Power
GABA	Gamma-Aminobutyric Acid
GHG	Greenhouse Gas Emissions
GID	Gastrointestinal Digestion
GLP-1	Glucagon-Like Peptide-1
GPx	Glutathione Peroxidase
GPR41	G Protein-Coupled Receptor 41
GPR43	G Protein-Coupled Receptor 43
GPR109A	G Protein-Coupled Receptor 109A
GSP	Glycosylated Serum Protein
HbA1c	Glycated Hemoglobin A1c
HCAR2	Hydroxycarboxylic Acid Receptor 2
HDL	High-Density Lipoprotein Cholesterol
HFD	High-Fat Diet
HOMA-IR	Homeostatic Model Assessment of Insulin Resistance
IL-1β	Interleukin-1 Beta
IL-6	Interleukin-6
IL-8	Interleukin-8
LAB	Lactic Acid Bacteria
LCA	Life Cycle Assessment
LDL	Low-Density Lipoprotein Cholesterol
LSF	Liquid-State Fermentation
MS	Multiple sclerosis
NCDs	Non-Communicable Diseases
NF-κB	Nuclear Factor Kappa B
Nrf2	Nuclear Factor Erythroid 2–Related Factor 2
PYY	Peptide YY
RCT	Randomized Controlled Trial
RFOs	Raffinose Family Oligosaccharides
ROS	Reactive Oxygen Species
SCFAs	Short-Chain Fatty Acids
SOD	Superoxide Dismutase
SSF	Solid-State Fermentation
TAC	Total Antioxidant Capacity
TFC	Total Flavonoid Content
TNF-α	Tumor Necrosis Factor Alpha
TPC	Total Polyphenol Content
